# Autologous Serum Eye Drops: A Systematic Review, Part I—Characteristics, Preparation, Storage, and Stability

**DOI:** 10.3390/bioengineering13101216

**Published:** 2026-10-19

**Authors:** Karolina Kucerova, Enkela Hrdlickova, Pavel Studeny, Lukas Balogh, Tor Paaske Utheim, Katerina Jirsova

**Affiliations:** 1 Institute of Biology and Medical Genetics, First Faculty of Medicine, Charles University and General University Hospital in Prague, 128 00 Prague, Czech Republic; karolina.kucerova@fnkv.cz (K.K.); lukas.balogh@lf1.cuni.cz (L.B.); 2 Ophthalmology Department of the Third Medical Faculty, University Hospital Kralovske Vinohrady, 100 34 Prague, Czech Republic; enkela.hrdlickova@fnkv.cz (E.H.); pavel.studeny@fnkv.cz (P.S.); 3 Department of Medical Biochemistry, Oslo University Hospital, 0424 Oslo, Norway; utheim2@gmail.com; 4 Department of Plastic and Reconstructive Surgery, Oslo University Hospital, 0372 Oslo, Norway; 5 Department of Ophthalmology, Sørlandet Hospital Trust, 4838 Arendal, Norway

**Keywords:** autologous serum, autologous serum eye drops, dry eye disease, growth factors, preparation, storage, stability

## Abstract

This two-part review provides a comprehensive overview of autologous serum eye drops (ASEDs), addressing their preparation (Part I) and clinical application in ocular surface diseases, particularly dry eye disease (DED) and persistent epithelial defects (PEDs) (Part II). Part I systematically reviews the current evidence on the characteristics, preparation, storage, stability, and microbiological safety of ASEDs. ASEDs contain multiple bioactive components, also present in tears, including growth factors, vitamins, cytokines, neurotrophic factors, antimicrobial proteins, and extracellular matrix-associated molecules, which may promote ocular surface homeostasis, epithelial regeneration, and wound healing. Particular attention is given to differences between natural tears and serum, as well as to disease-related changes in serum that may influence the biological properties and therapeutic efficacy of ASEDs. Preparation protocols vary considerably among studies and clinical centers, including variation in blood collection volume, clotting time, centrifugation parameters, dilution, filtration, storage temperature, and container type. These factors may influence the final concentration and stability of biologically active components and may also affect microbiological safety. Database searches identified 653 records, of which 288 were screened and 200 reports were assessed for eligibility; 75 studies met the inclusion criteria. After inclusion of eligible studies from the personal literature database and removal of overlap between the two sources, 198 unique studies were included in the systematic review. Although ASEDs are widely used as biologically active tear substitutes, further standardization of preparation, storage, and handling protocols is needed to improve product consistency and safety and to facilitate comparison and reproducibility of clinical outcomes.

## 1. Introduction

For many decades, human blood products have been used in the treatment of a wide range of diseases [1,2]. Blood is readily accessible and some of its components—serum, plasma, and platelets—contain a number of protein and non-protein compounds effective in wound healing and regeneration [3,4]. Their combined biological activity may support tissue homeostasis, while adverse reactions are generally minimal. Human serum-derived products, including autologous serum, allogeneic serum, and umbilical cord serum, are among the most widely used biological therapies for accelerating ocular surface epithelial wound healing [3,4,5].

Autologous serum eye drops (ASEDs) are administered predominantly to patients with severe dry eye disease (DED) [6,7]. They have been used in a range of ocular surface diseases, including Sjögren syndrome-associated DED [8,9], ocular graft-versus-host disease (oGVHD) [10,11], Stevens–Johnson syndrome (SJS), ocular cicatricial pemphigoid, a manifestation of mucous membrane pemphigoid [12], superior limbic keratoconjunctivitis [13], neurotrophic keratopathy [14], and limbal stem cell deficiency [15].

Another major indication for ASED treatment is persistent epithelial defect (PED) related to various causes (infectious, postoperative, other trauma to the ocular surface) [16,17,18,19,20], or recurrent corneal erosion [21,22,23]. ASEDs are also used as supportive treatment in ocular surface reconstruction [24,25].

Despite their widespread use, substantial variability remains in ASED preparation, processing, composition, storage, stability, and handling among published studies and clinical settings. These differences may affect the concentration and stability of bioactive components and the microbiological safety and consistency of the final product. A systematic synthesis of the available literature is therefore needed to summarize current knowledge and identify areas requiring greater standardization.

Given the breadth of the topic, the review is divided into two complementary parts, Part I and Part II. The objective of Part I was to summarize the current knowledge on the characteristics, preparation, storage, stability, and microbiological safety of ASED, with particular attention to factors that may influence their biological properties, safety, and consistency. Part II addresses their clinical application, including therapeutic indications, administration, clinical outcomes, and safety. This division was chosen to separate product-related and methodological aspects from clinical evidence and to allow each topic to be discussed in sufficient detail.

## 2. Materials and Methods

### 2.1. Literature Search

The literature search and study selection process were conducted and reported in accordance with the PRISMA 2020 guidelines. To prepare this review, an extensive literature search was performed, and data from both clinical and experimental studies were analyzed. Particular care was taken to evaluate original studies rather than secondary citations. Articles concerning ASEDs, their composition, preparation, storage, stability, and clinical use were obtained from the PubMed and Web of Science databases. PubMed was selected as a major biomedical database covering the clinical and experimental literature relevant to ASEDs, while Web of Science was used as a complementary multidisciplinary source. No additional bibliographic databases were systematically searched for the present review. In addition, a personal literature database was developed and continuously updated over approximately 25 years through ongoing searches for ASED-related publications. It therefore included relevant articles obtained from different literature sources, irrespective of the database in which they were originally retrieved. It contains 621 records related to ASEDs and an additional 95 records related to DED. Publications retrieved from the personal literature database were screened as an additional source of potentially eligible studies and were assessed according to the same eligibility criteria as records identified through the systematic database searches.

To provide a structured and reproducible search of the current literature, systematic searches of PubMed and Web of Science were performed using predefined search strategies. The primary PubMed search strategy combined “autologous serum” and “eye” in the Title/Abstract fields and was restricted to human studies. Additional PubMed searches used predefined terms related to the main topics addressed in Part I, including history, preparation, centrifugation, dilution, serum concentration, storage, freezing, preservation, microbiological contamination, stability, composition, growth factors, vitamins, cytokines, effects, reviews, disease, dry eye, and ocular surface. The results of the individual searches were combined using the OR operator to identify unique records. Web of Science was searched to identify additional relevant publications. 

Unless otherwise stated, the included studies were performed in humans or using human cells. Relevant publications were grouped according to the main topics addressed in Part I, including ASED preparation and processing, composition and biological components, differences between serum and tears, storage and preservation, stability, and microbiological safety. Statistical significance was interpreted according to the thresholds and adjustments reported in each original study.

### 2.2. Eligibility Criteria

Publications were considered eligible for inclusion in the systematic review if they addressed ASEDs and provided data or directly relevant evidence related to the objectives of Part I, including ASED characteristics, composition and biological components, preparation and processing, storage and preservation, stability, or microbiological safety. Studies comparing serum with natural tears or evaluating tear- and serum-related biological components were also eligible when directly relevant to the characteristics or biological properties of ASEDs. Both clinical and experimental studies involving humans, human serum, tears, or human cells were considered eligible.

Studies were excluded if they did not address ASEDs or did not provide data relevant to the objectives of Part I; focused exclusively on clinical efficacy or therapeutic outcomes without providing relevant information on ASED characteristics, preparation, composition, storage, stability, or safety; investigated only other blood-derived or biological eye-drop preparations without separately reported ASED data; or were performed exclusively in animals. Animal studies cited to explain biological mechanisms were used as contextual references and were not counted among the included studies. Review articles, guidelines, and other secondary sources were used where directly relevant to the predefined thematic domains, particularly for contextual interpretation and identification of relevant original publications. Given the broad descriptive scope of the review and the substantial methodological heterogeneity of the included literature, which comprised clinical, observational, experimental, laboratory, and methodological studies addressing different aspects of ASED characteristics and preparation, a formal risk-of-bias assessment was not considered appropriate. The evidence was therefore synthesized descriptively according to the predefined thematic domains of the review.

### 2.3. Study Selection

The identified records were screened independently by KJ and LB based on their titles and abstracts for relevance to the objectives of Part I. Disagreements between the reviewers were resolved by discussion and consensus. Potentially relevant articles were subsequently assessed in full text by KJ and LB according to the predefined eligibility criteria. Publications meeting these criteria were included in the systematic review. Overlap between the systematic database searches and the personal literature database was identified by comparison of the bibliographic information of individual publications, and duplicate records were removed before final inclusion.

### 2.4. Data Extraction and Synthesis

KJ and KK independently extracted relevant data and assigned each study to one or more predefined thematic areas. The extracted information was subsequently cross-checked, and any discrepancies were resolved by discussion and consensus. Data of interest included study characteristics and findings related to ASED composition and biological components, preparation and processing procedures, differences between serum and tears, storage and preservation conditions, stability, and microbiological safety. All results relevant to these predefined domains were considered for data extraction. Where reported, data on the historical development of ASEDs and specific parameters, including the composition and concentration of biologically active components in serum and tears in healthy individuals and patients with ocular surface diseases; blood collection volume; centrifugation parameters; dilution and filtration procedures; ASED concentration; storage conditions and duration; and stability of biological components, were extracted. The extracted information was organized according to the main topics addressed in Part I and synthesized descriptively because of the methodological and procedural heterogeneity among the included publications. Where appropriate, extracted data were summarized in tables according to the main thematic areas of the review.

## 3. Results of the Literature Search

**Figure 1:**
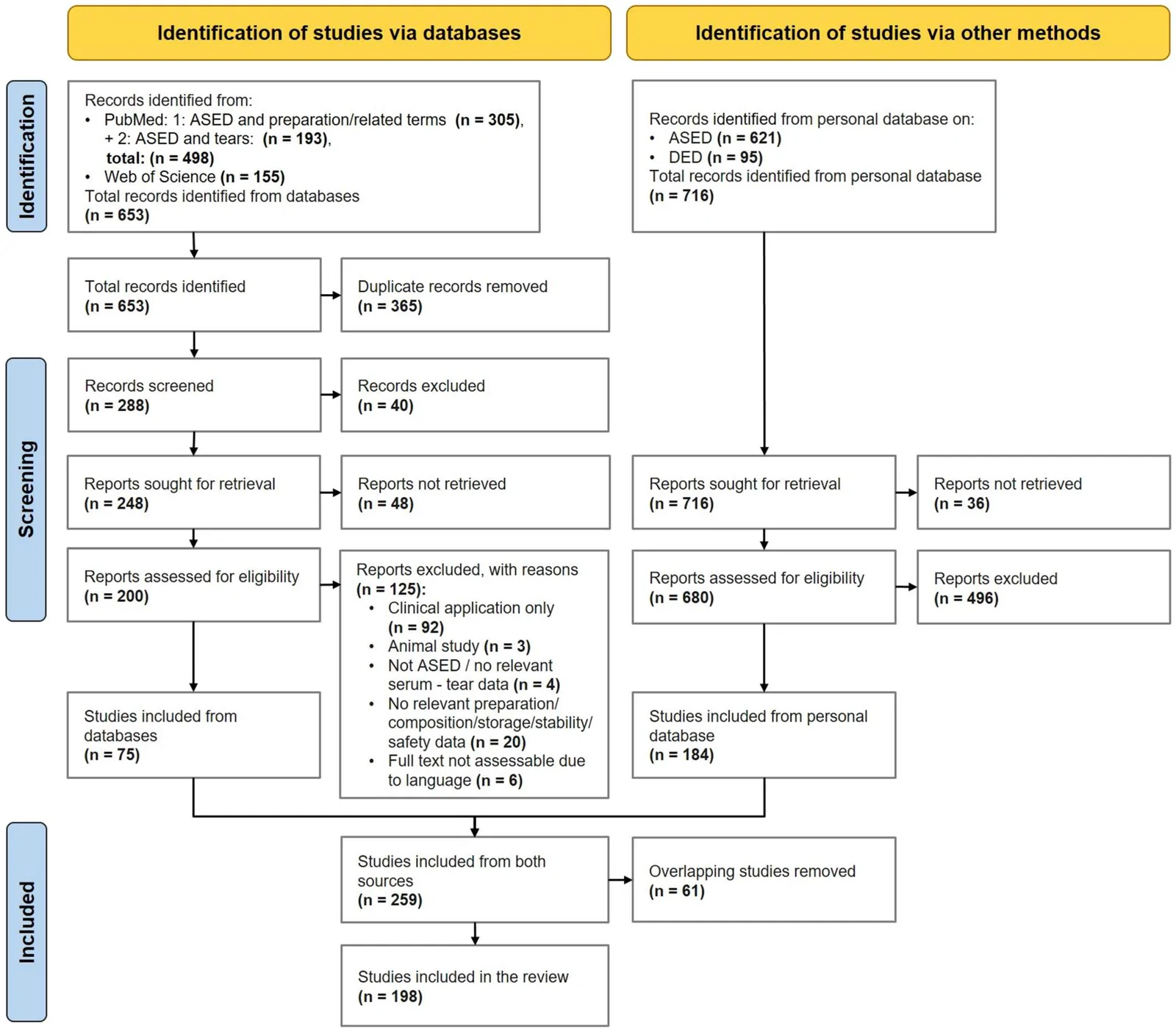
PRISMA 2020 flow diagram of study selection.

The database searches identified 653 records, including 498 records from PubMed and 155 from Web of Science. After removal of 365 duplicate and overlapping records, 288 records were screened by title and abstract, of which 40 were excluded. Full texts were sought for 248 reports; 48 reports could not be retrieved, and 200 reports were assessed for eligibility. Following full-text assessment, 125 reports were excluded, resulting in 75 studies included from the database searches. In addition, 716 records were identified from the personal literature database, of which 680 were assessed for eligibility and 184 met the inclusion criteria. Together, the two sources yielded 259 potentially included studies before removal of overlap. After removal of studies overlapping between the two sources (n = 61), a total of 198 unique studies were included in the systematic review. The study selection process is presented in the PRISMA flow diagram, Figure 1. The complete search strategies and search dates are provided in Supplementary Tables S1 and S2. In addition, a PubMed search using the keywords “autologous serum”, “eye”, and “drops” showed an overall increase in the number of publications over time, with peaks in publication activity in 2008, 2016, and 2025, reflecting sustained research activity and clinical interest in ASED.

## 4. History and Development of Autologous Serum Eye Drops

Autologous serum for the treatment of ocular surface diseases (SJS, alkali burns) was first applied using a mobile ocular perfusion pump in 1975 by Ralph et al., with some success [26]. Later, in 1984, Fox et al. showed a beneficial effect of autologous serum by treating patients with DED and Sjögren syndrome for three weeks, markedly improving Rose Bengal staining scores [27].

**Figure 2:**
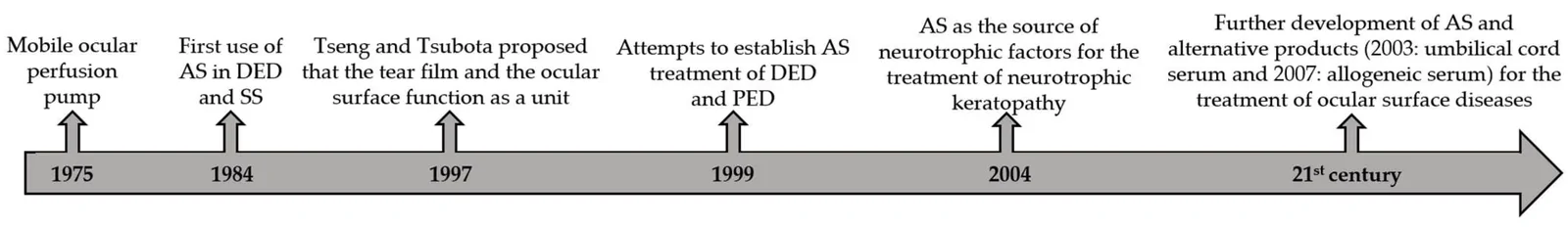
History and development of autologous serum and its use in therapeutic applications: 1975: [26], 1984: [27], 1997: [28], 1999: [6], 1999: [16], 2004: [14], 21st century: 2003 [29], 2007 [30]. AS: autologous serum, ASED: autologous serum eye drops, DED: dry eye disease, PED: persistent epithelial defect, SS: Sjögren syndrome.

In 1997, Tseng and Tsubota hypothesized that autologous serum could replenish epithelial modulating factors reduced in tear deficiency, including epidermal growth factor (EGF), transforming growth factor-beta (TGF-β) and vitamin A [28]. In 1999, Tsubota et al. published two studies demonstrating efficacy and safety of ASED in the treatment of DED and PED [6,16]. In 2004, Matsumoto et al. demonstrated the presence of neurotrophic components in ASEDs and their potential beneficial effects [14]. Key milestones in the development of ASEDs are summarized in Figure 2.

At the beginning of the 21st century, alternative products, including umbilical cord serum [29] and allogeneic serum [30], were introduced, particularly for patients unsuitable for venipuncture or at risk during ASED treatment [30].

## 5. Autologous Serum Composition and Effects

### 5.1. Composition of Autologous Serum and Tears

The rationale for using ASEDs is based partly on their compositional similarities to natural tears; they contain electrolytes, functional and structural proteins, lipids, and carbohydrates [31,32]. The biomechanical, biochemical, and molecular properties (osmolarity, pH, composition) of tears and serum are very similar, although the concentrations of individual components may differ substantially (Table 1, Table 2, Table 3 and Table 4) [31]. However, it should be emphasized that measured concentrations of specific compounds can be affected by the method used for tear collection (Schirmer test or capillary test) and by the method used for analysis [33,34].

ASEDs contain growth factors, cytokines, chemokines, and vitamins with pro-epithelialization, anti-inflammatory, antioxidative, and antinociceptive properties, providing a biological rationale for their potential role in ocular surface homeostasis and wound healing [6,96]. As an autologous product, ASEDs are non-immunogenic and non-allergenic and are generally well tolerated [17,31]. ASEDs naturally moisten the ocular surface and thus may help reduce tear hyperosmolarity associated with DED. Unlike preservative-containing artificial tears, ASEDs do not contain preservatives that may adversely affect ocular surface cells and cell membrane integrity [17], and thus their use is associated with minimal adverse reactions.

Serum contains approximately tenfold higher total protein concentrations than tears, and the concentrations of individual components vary considerably between the two fluids [35,41,44]. The major tear proteins are lactoferrin, lysozyme, and lipocalin, whereas the main proteins in serum are albumin and immunoglobulin G (IgG) [32,42,46,97]. Tear proteins maintain surface tension and support hydration and homeostasis of the ocular surface epithelium. Lactoferrin (also known as lactotransferrin) has strong antibacterial, antiviral, and antioxidative properties, and may reduce pro-inflammatory mediators associated with DED and protect the corneal epithelium against UV irradiation [98,99]. Lipocalin is the main carrier of lipids in human tears ensuring wetting and stabilizing of the ocular surface. It is thought to clear the ocular surface by sloughing cellular debris [100]. Compared with serum, lactoferrin levels are higher in tears [101]. Lacritin, a protein with mitogenic features, promotes basal tearing and survival of corneal epithelial cells [100]. Another protein present in tears, albumin, stabilizes the tear film and may be involved in inhibition of apoptosis [102].

Tears contain factors involved in epithelial homeostasis and repair, including fibronectin, EGF [103], platelet-derived growth factor (PDGF) [64], TGF-β, hepatocyte growth factor (HGF) [74], vitamins A, and E, and hyaluronic acid, all of which are also present in serum [68,69,104,105,106,107].

Fibronectin in tears most likely originates from blood plasma [50]. It enhances the migration of epithelial cells and their attachment to the epithelial defect [108]. EGF inhibits epithelial cell terminal differentiation and modulates proliferation in a dose-dependent manner: a considerable increase in proliferation of human corneal epithelial cells was found at concentrations > 0.1 ng/mL [109,110], but less markedly at concentrations exceeding 100 ng/mL [109]. The production of EGF can be increased by mechanical irritation of the cornea (e.g., caused by certain types of contact lenses) [57]. EGF also stimulates migration of epithelial cells, particularly at locations with lower cell density, and thus accelerates re-epithelization and wound healing. EGF drives the generation of cells with a conjunctival epithelial phenotype (compared with corneal epithelial cells), i.e., increases proliferation and the expression of goblet cell markers, as shown on animal [111], and human cells [112,113]. It acts against apoptosis and stimulates fibronectin synthesis [73,114].

TGF-β has been shown to play a pleiotropic role on the ocular surface, suppressing autoimmune responses under physiological conditions while enhancing the inflammatory response under stress conditions [70,71,115,116]. TGF-β is generally thought to inhibit proliferation and induce normal differentiation of epithelial cells. It modulates the effects of EGF, keratinocyte growth factor, HGF, and fibronectin. It also stimulates proliferation of stromal cells and the secretion of matrix metalloproteinases (MMP) MMP-1, -3, -9, -10, -11, and -13 by human corneal epithelial cells thus modulating later responses during wound closure [73,116,117]. The concentration of TGF-β1 in tears is somewhat higher compared to TGF-β2 [35,65,118]. Due to concerns about the potential antiproliferative effects and suppression of ocular surface epithelial wound healing associated with concentrated serum, which contains approximately fivefold higher concentrations of TGF-β1 than tears [65,67], Tsubota et al. began to dilute serum with saline 1:4 to obtain a concentration of TGF-β1 closer to that found in natural tears [6,35]. Since then, a 20% serum dilution has been used in numerous subsequent studies and has become the de facto standard concentration [7,119,120,121,122,123,124].

Besides pro-epithelial factors in tears, there are other growth factors participating in ocular surface stability. These are neurotrophic factors which include nerve growth factor (NGF), brain-derived neurotrophic factor, and neurotrophin-3 and -4 as well as insulin-like growth factor (IGF), and substance P, which contribute to neural proliferation, differentiation, and wound healing [75,76,86]. These growth factors are also present in serum [60,77,78]. It has been shown that the concentrations of NGF, IGF-1 and substance P in serum are higher than in tears [14,75]. Among the neurotrophic factors present in tears, NGF in particular has been shown to play an important role in the ocular surface epithelium, increasing the number of goblet cell numbers and mucin (MUC5AC) synthesis [14,73,110,125]. Beyond the analysis of individual bioactive factors, more recent proteomic studies have provided a broader characterization of the protein composition of autologous serum [126].

Other essential components of tears and serum are vitamins A, B (B1, B2, B3, B5 and B9), C, D, and E [48,79,84,127]. Vitamin C and B3 levels are significantly higher in tears than in serum [79,81,82]. Vitamin E concentrations are significantly higher in serum than in tears [79]. Vitamin A is abundant in serum and occurs at much lower or undetectable concentrations in tears [48,79,127]. In contrast, total 25-hydroxyvitamin D concentrations were significantly higher in tears than in serum [83]. These vitamins contribute to ocular surface health through different mechanisms: vitamin A supports goblet cell viability [128], vitamin D has anti-inflammatory and immunoregulatory functions [129], and vitamin E contributes to epithelial differentiation and antioxidant defense [130]. Vitamin D is of particular importance, as vitamin D deficiency is considered a risk factor for the development of dry eye disease [131,132]. Reduced vitamin D levels in tears and serum have also been reported in patients with DED, and vitamin D is considered one of the most potent antinociceptive factors in tears [84].

Apart from proteins, tear lipids play a vital role in maintaining homeostasis of the ocular surface. The tear-lipid film forms a layer spread over the aqueous layer on the ocular surface and thus retards evaporation, provides a smooth optical surface, and acts against invasion and colonization by microorganisms [133,134]. Many lipid components have been detected in human serum [135], and they can replenish the pathological loss of lipids on the ocular surface [13].

Tear fluid also exhibits strong antimicrobial activity, which protects the ocular surface against bacterial, viral, fungal, and protozoal infections. The main tear defense proteins, which are also present in serum, include immunoglobulins IgG and IgA [66], lysozyme and lactoferrin [35,42,43,45]. Lactoferrin is present at very low concentrations in serum [89], where some of its antimicrobial functions are partially compensated by transferrin [35,42,90]. IgE is present at lower levels in the tears of healthy individuals; it is predominantly found in the serum, but in patients with allergic keratoconjunctivitis it is significantly higher in tears [87]. Alpha-2-macroglobulin, which serves as a non-specific inhibitor of proteolytic enzymes, supports extracellular matrix production [88,136].

Antioxidants present in both serum and tears include lactoferrin, vitamin C (both discussed above), uric acid, glutathione, tyrosine, cysteine, and selenoprotein P, which participates in selenium transport [32,35,54,91,94]. The regulation of inflammation, immune responses and pain on the ocular surface is orchestrated by numerous cytokines and interleukins (IL) present in physiological and pathological tear fluid [33,58,62,84,137]. Most of these factors are also present in serum [72]. The main anti-inflammatory factor present in both tears and serum is IL-10, while IL-1β, IL-6, IL-8, IL-17A, tumor necrosis factor alpha (TNF-α), and interferon-γ (IFN-γ) are among the main pro-inflammatory factors [70,138,139]. Sustained presence of inflammatory factors on the ocular surface may result in the disruption of corneal barrier function and homeostasis, which can contribute to DED symptoms [140]. Particular caution is warranted when using high-concentration ASEDs (including 100% serum) or ASEDs prepared from patients with autoimmune or systemic diseases, as these conditions are associated with elevated levels of pro-inflammatory cytokines. Whereas IL-13 supports ocular surface homeostasis by promoting goblet cell differentiation, IFN-γ induces squamous metaplasia and apoptosis, and IL-17 impairs epithelial barrier function [141].

ASEDs were the second most effective topical ophthalmic therapy after artificial tears in patients with severe ocular pain, with a response rate of 55% [96]. The analgesic effect of ASEDs may be relevant for patients with DED, particularly those with corneal neuropathic pain [142,143] or postoperative ocular pain [144,145]. Taken together, the biological activities of individual serum components support the therapeutic rationale for ASEDs, while their clinical effect reflects the combined action of multiple factors.

### 5.2. Differences in Tear Composition Based on Subjects’ Health

Considering treatment with ASEDs, it is necessary to emphasize that not only the composition of tears, but also the serum composition of healthy individuals and patients with ocular or systemic diseases may differ (Table 1, Table 2, Table 3 and Table 4), and thus influence the therapy. Compared with healthy tears, DED tears are characterized by significant hyperosmolarity [36,38,39], although some studies found similar mean osmolarity in healthy controls and patients with DED [37,40]. DED tears show decreased concentrations of lysozyme, lactoferrin, and lipocalin, the most abundant tear proteins [55,146,147]. Among these proteins, a significant decrease has been repeatedly reported for lactoferrin [42,148], with particularly low levels detected in tears from patients with DED associated with Sjögren syndrome, SJS, or oGVHD [45,55,149]. Finally, using a meta-analytic approach, it has been suggested that lactoferrin decrease in DED tears may serve as a good DED diagnostic biomarker [150]. A significant decrease in tear EGF concentrations (*p* < 0.005) was observed in patients with Sjögren syndrome-associated DED compared with those in healthy controls [56,151]. A similar finding (lower levels of EGF in tears compared to control tears) was reported in patients with chronic oGVHD [59].

A significant increase in the pro-inflammatory factors IL-1α, IL-6, IL-8, TNF-α compared with control tears from healthy individuals was found in Sjögren syndrome [58]. Using the same controls, patients with non-Sjögren DED showed a significant increase in IL-8 and TNF-α only [58]. Almost identical results (increased expression of pro-inflammatory IL-1α, IL-6, IL-8, TNF-α) in Sjögren syndrome patients were found previously on conjunctival epithelial cells [56].

Characteristic elevation of pro-inflammatory factors, which partially differ from typical DED tear factors profile (e.g., granulocyte–macrophage colony-stimulating factor, IL-2, IL-1β, basic fibroblast growth factor and IL-17A) together with downregulation of antifibrotic proteins, such as IFN-γ-induced protein 10, IFN-γ, TNF-α was found in the tears of patients with chronic SJS [138].

Elevated tear-soluble factors with pronociceptive potential (IL-1β, IL-17A, MMP-9, and MMP-10) and VEGF-B, together with reduced antinociceptive factors (VEGF-A, IL-2, and vitamin D), were observed in evaporative DED, and these changes were significantly associated with DED severity [84]. The proteins reported to be increased or decreased in DED tears were reviewed by Karnati et al. [100].

### 5.3. Differences in Serum Composition Based on Subjects’ Health

Changes observed in the DED tear composition of patients may, but do not necessarily, reflect changes in patients’ serum. Differences in serum composition have been investigated between healthy individuals and patients with DED [60], between patients with and without systemic disease [152], and between inactive and active Sjögren syndrome [153]. All these alterations in serum composition may influence the therapeutic effect of ASEDs.

No statistically significant differences in the concentrations of epitheliotropic and neurotrophic factors were observed between healthy controls and idiopathic DED patients [60]. Similarly, reactive oxygen species and total reactive antioxidant potential did not differ between 50% ASED prepared from patients with DED and healthy controls [154]. There was also no difference in epithelialization-supporting factors (EGF, TGF-β1/β2, and fibronectin) in 20% ASED from patients with non-autoimmune DED and those with chronic SJS [68].

When comparing patients with inactive and active forms of Sjögren syndrome, significantly higher serum levels of TGF-β1 and hyaluronic acid were found in the latter group [153]. Levy et al. (2019) showed significantly higher serum EGF concentrations in patients with DED of various etiologies who were non-responders to ASED therapy compared with responders, with response defined by improvement in the ocular surface disease index (OSDI) score and/or corneal fluorescein staining [49]. Significantly higher levels of EGF were found in patients with localized ocular surface DED compared to patients with DED associated with systemic disease. The levels of fibronectin, IL-8, IL-17, TNF-α or vitamin A did not differ [152]. Although OSDI improved significantly overall after six weeks of treatment, symptom reduction was numerically greater in patients with localized disease, but the between-group difference was not significant [152]. It has also been suggested that a decrease in the concentration of epitheliotropic factors (EGF, PDGF, TGF-β) in serum, as occurs in patients with diseases affecting other organs, such as chronic renal failure, may reduce the therapeutic efficacy of ASEDs [155]. Taken together, these findings suggest that the relationship between serum EGF concentration and ASED efficacy is complex and that EGF alone cannot yet be considered a reliable predictor of treatment response. Future studies with larger cohorts are needed to determine whether serum EGF levels may serve as a predictor of ASED treatment efficacy.

Similarly, the concentration of pro-inflammatory compounds can be markedly higher in serum of DED patients with autoimmune or systemic disease [9], or with the active form of the disease [153]. Patients with active Sjögren syndrome have significantly higher levels of pro-inflammatory factors in serum (IL-1β, IL-6, and TNF-α) compared to patients with inactive disease [153].

## 6. Preparation of Autologous Serum Eye Drops

### 6.1. Blood Collection, Clotting and Centrifugation

**Figure 3:**
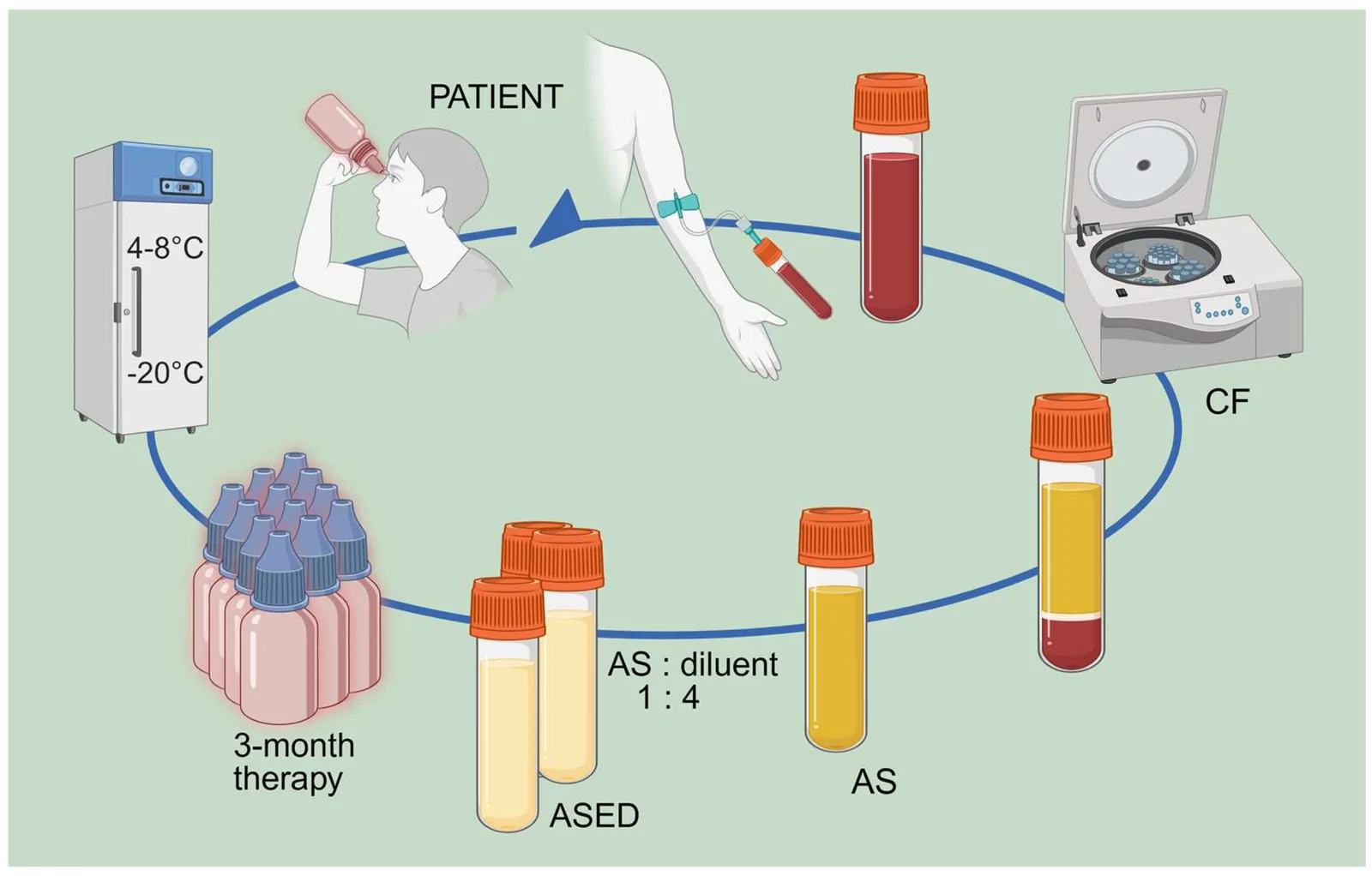
Preparation of autologous serum eye drops (ASED). Peripheral venous blood is collected from the patient, typically 40–60 mL for approximately 3 months of treatment. After clotting at room temperature for at least 2 h (or up to 2 days at 4 °C), blood is centrifuged at 1500–3300× *g* for 10–15 min. The resulting serum is processed under sterile conditions and may be diluted with sterile saline, balanced salt solution, or sodium hyaluronate, most commonly to 20% or 50% concentrations. ASED are dispensed into sterile, UV-protective vials; the vial in current use is stored at 4–8 °C, while the remaining vials are frozen at approximately −20 °C for up to 3 months. Figure created in BioRender by authors (2026), https://BioRender.com/vc1q5xa.

Although ASEDs have been used for several decades, their production and application remain non-standardized [31,156,157]. Legislation and recommendations differ between countries, including within Europe [31]. In the USA, there is no single standardized federal protocol specifically for ASED preparation; applicable requirements depend on how and where ASEDs are prepared and may include federal compounding provisions, state regulations, and institutional policies [7,158]. Since all steps of ASED preparation and storage (Figure 3), including clotting time, centrifugation time and force, dilution procedures, and storage conditions (time and temperature) can affect the concentration of active substances [61], procedural variability may jeopardize product consistency. Standardized protocols are therefore important for quality control and clinical implementation [159,160].

The results of numerous clinical studies suggest that ASEDs remain clinically effective despite considerable variability in preparation protocols. ASEDs are usually produced in pharmacies, eye banks, or blood services equipped with the appropriate facilities [7,161]. Blood is collected by venipuncture into serum collection tubes or, for larger volumes, sterile blood collection bags without anticoagulant; collection volumes range from 40 to 450 mL [123,162,163]. Generally, 40–60 mL of blood is sufficient for 3-month therapy using 20% ASED (up to 10 times daily) or 50% ASED (4 times daily) [6,163].

Larger volumes of blood are obtained at transfusion departments [164]. Contraindications to high-volume sampling (above 100 mL) include anemia (hemoglobin < 11 g/dL), severe cardiovascular disease or active infection [31,165]. The blood sample should be tested for hepatitis B/C and HIV-1/2, with additional screening according to national legislation for syphilis, human T-lymphotropic virus I/II, cytomegalovirus, West Nile virus, and Chagas’ disease [31,156,162,164]. Although positive serology is not an absolute contraindication to autologous use, patients with blood-borne infections are frequently excluded from standard manufacturing protocols because of biosafety requirements, the risk of cross-contamination, and institutional or regulatory policies [31,165]. On the other hand, handling infected blood may increase the risk of occupational exposure for personnel preparing ASEDs [166,167].

Tubes with blood should be left in a vertical position at room temperature for a minimum of 2 h or at 4 °C up to 2 days, to facilitate coagulation and improve serum yield [123]. It has been shown that increased clotting time from 20 min to 2 h led to significant elevation of EGF, TGF-β1 and HGF levels [61]. In anticoagulated patients, insufficient clotting may cause serum gelation, and a resting time of at least 60 min has been recommended [168].

Centrifugation is usually performed at 1500–3300× *g* for 10–15 min [9,31,120,123,153,169,170]. The parameters should preferably be reported as relative centrifugal force (× *g*) rather than rpm, as the centrifugal force at a given rpm depends on the rotor radius [31]. Compared with 500× *g* for 5 min, centrifugation at 3000× *g* for 15 min decreased TGF-β1 levels, whereas EGF and vitamin A levels increased [61]. However, no significant differences in any of the main ASED growth factors were observed between centrifugation at 2500× *g* for 10 min and 3000× *g* for 15 min, suggesting that more intensive centrifugation does not necessarily increase growth factor concentrations [171].

### 6.2. Dilution and Filtration

After centrifugation, ASEDs are prepared under strict aseptic conditions in a biosafety cabinet in order to avoid cross-contamination. The blood of individuals should be treated separately. Clear serum with no blood clot may then be transferred to a sterile container (vials, eye dropper bottles, daily-dose dropper bottles) [123,169,172,173,174]. Some studies recommend ASED filtration using 0.2–0.45 μm pore size filters. The 0.2 μm filter is able to retain blood clot debris and bacteria without changing the serum peptide profile or decreasing the concentration of the heaviest peptides, as demonstrated for TGF-β1 [169]. Alternatively, low protein-binding filters can be used [175].

Apart from sterile saline used as a solvent, no preservatives, stabilizers, or additives are added to ASED in the vast majority of ASED preparation protocols [6,14,165,170]. Less frequently, serum is diluted with balanced salt solution [61,105,176]. Alternatively, sodium hyaluronate eye drops have been used, generating a product showing positive results in clinical trials [9,123,177]. More recently, cyclosporine A has also been shown to be a safe diluent for ASED in vitro, providing additional anti-inflammatory properties [178]. It has been demonstrated that ASEDs with sodium hyaluronate are better tolerated than ASEDs diluted with saline and that sodium hyaluronate adheres well to the mucin fraction, thereby increasing the stability and prolonging the activity of ASED on the ocular surface [123].

ASEDs are generally applied at 20% of the original concentration [14,16,21,179], less commonly at 50% [163,165], or undiluted [19]. However, use of the undiluted serum may necessitate more frequent blood draws if ASED are used for a longer period of time [19]. Dilution of ASED to 20% provides the benefit of obtaining a larger volume of ASED per blood collection [17,180], and its efficacy in DED treatment has been confirmed in numerous clinical trials [122]. Although 20% ASED is the most frequently used concentration, current evidence does not demonstrate its superiority over higher concentrations. Its widespread use appears to reflect long-standing clinical practice and is also favored by the pragmatic advantage of obtaining a larger treatment volume from a single blood collection.

Antibiotics may be added to ASEDs, particularly those with low epithelial toxicity, e.g., chloramphenicol or ceftazidime [17], with the aim of increasing the antimicrobial potential of ASEDs. Under experimental conditions, levofloxacin significantly decreases the number of contaminated samples [181]. Most protocols avoid preservatives, partly because serum contains antimicrobial components that may help protect both the eye drops during storage and the ocular surface after the application. Additionally, exogenous substances are not preferentially added to preserve the native character of ASEDs [17,18].

ASEDs are usually dispensed into 2–5 mL sterile, UV-protective vials, as vitamin A is degraded by UV light [6,21,169], and appropriately labelled [157,182].

Large volumes of serum (diluted product) can be distributed in eye drop bottles using a pump [183]. The use of single-day bottles (daily-dose dropper bottles), which reduces the probability of ASED contamination due to repeated manipulation, is another option [169,174,182,184]. However, conventional dropper bottles showed the highest patient acceptance and were considered more suitable for patients with visual or manual impairments [174,184].

### 6.3. Control Examination and Microbiology

ASEDs can be administered after negative microbiological testing [170,185]. Under controlled experimental or validated manufacturing conditions, no microbial contamination was detected [186]. This was demonstrated with refrigerated undiluted ASEDs and single-use applicators, as well as in 2297 batches manufactured using a closed system with 0.22 µm sterile filtration [162,187,188]. However, microbial contamination has also been reported during routine patient use. In clinical practice, the most commonly used storage protocol involves keeping one vial in a refrigerator for up to one week, while the remaining vials are stored at −20 °C in a freezer. Repeated handling of the in-use vial, particularly improper cap handling or touching the dropper tip, may increase the risk of microbial contamination [173,181].

In one study, refrigerated ASED dropper bottles remained sterile during the first four days; after one week, 7.5% of samples showed bacterial contamination, with no fungal contamination observed [189]. Another study detected bacterial contamination in approximately 10% of samples collected before and after 24 h of ASED use [190]. A relatively high contamination rate of 55% was reported in ASED samples returned by patients 30 days after preparation [191]. Single-day bottles showed an overall contamination rate of approximately 6% in samples collected before use and after 24 h of use [173]. The container system also appears to be important: contamination occurred in only 2.1% of containers equipped with a sterilizing filter after up to four weeks of home use, compared with 29% of conventional containers after seven days [192], while a one-way-valve system maintained sterility of the bottle contents in 103/104 bottles during extended patient use [184]. Overall, these findings underscore that controlled preparation and storage, filtration, and single-use or protected dispensing systems mitigate the risk of microbial contamination, whereas prolonged use and repeated handling by patients increase this risk. The most frequently identified contaminants in ASED were coagulase-negative staphylococci, particularly *Staphylococcus epidermidis*, as well as *Corynebacterium* spp. and *Micrococcus* spp.; occasionally, more pathogenic species (*Staphylococcus aureus*, *Streptococcus viridans*, *Klebsiella pneumoniae*, *Pseudomonas aeruginosa*) or fungi (e.g., *Aspergillus* spp.) were also isolated [173,181,190,191]. Because the studies differed in sampling units, storage and use duration, dispensing systems, and microbiological methods, the reported contamination rates are not directly comparable (Supplementary Table S3).

## 7. Autologous Serum: Storage and Stability

Storage is an important determinant of the biochemical stability of ASEDs and may influence the preservation of their biological properties and thus their quality. Quality-control testing should be considered a separate priority for standardization and reporting. Potential parameters may include visual appearance, sterility, residual cellular contamination, and selected biochemical markers [162], although universally accepted ASED-specific specifications have not yet been established. The effects of storage temperature and duration on the stability of major bioactive components in ASEDs are summarized in Table 5. Most available studies assess stability by measuring the concentration of individual bioactive components, typically using immunoassays such as ELISA. Maintenance of the measured concentration indicates that the immunoreactive protein remains detectable but does not necessarily demonstrate retained biological activity. Direct functional data after storage is available only in a limited number of studies and is therefore discussed separately where available. The results summarized below should therefore be interpreted primarily as evidence of biochemical stability.

In clinical practice, a commonly used storage protocol is to keep the vial in use in a refrigerator at 4–8 °C for one week (or for one day in some protocols) [19], while the remaining vials are stored frozen at −20 °C for up to 3 months [31,170,179]. For optimal ASED quality, the stability of these bioactive components under both storage conditions should be considered; numerous studies have evaluated changes in the measured concentrations of epitheliotropic and neurotrophic factors during storage [6,16,48,63,68,105,123,169,193]. Some studies have reported longer-term storage, with the stability of individual components varying. The measured concentrations of EGF and TGF-β remained stable for up to one month at 4–8 °C [6,16]. At −20 °C, TGF-β1/2 levels decreased after one month, whereas fibronectin remained stable for up to three months [68]. Other studies reported stability of selected components during even longer frozen storage, for up to six or seven months [63,193], Table 5. The oxidative balance of 50% ASEDs remained stable during storage at −20 °C, with no significant changes in reactive oxygen species or total reactive antioxidant potential after 15 or 30 days [154]. However, most stability studies were performed at −20 °C, whereas household freezers typically operate at approximately −15 °C to −18 °C. The clinical relevance of this difference remains unclear.

For ASEDs intended for the treatment of neurotrophic keratopathy and ocular surface diseases, in which neurotrophic factors play an important role, it is necessary to consider that key factors, such as substance P, IGF-1, NGF, and calcitonin gene-related peptide, degrade significantly faster than other tested growth factors, Table 5 [63,169]. In contrast, insulin levels in ASEDs remain stable after one month of storage at −20 °C [191].

As an alternative method for ASEDs preservation, lyophilization has also been investigated. After reconstitution, 50% lyophilized ASEDs showed growth factor concentrations and in vitro biological effects on human ocular surface cell growth comparable to fresh serum, with stability for up to one month at 4 °C [194].

The costs of ASEDs vary substantially between providers. A 2018 review reported costs of approximately USD 175–250 for a two-month supply of ASEDs in the United States [195]. 

## 8. Discussion

ASEDs represent a biologically active tear substitute whose therapeutic potential is related to their complex composition and similarity to natural tears. In contrast to conventional artificial tears, ASEDs provide not only lubrication but also epitheliotropic, neurotrophic, anti-inflammatory, antimicrobial, and antioxidative components that may support ocular surface regeneration and homeostasis [31,196].

Variations in clotting time, centrifugation parameters, dilution, filtration, and storage conditions may influence the concentration and stability of growth factors, vitamins, cytokines, and other bioactive molecules [9,31]. Despite this variability in composition, there is currently limited evidence that variations in preparation protocols result in clinically meaningful differences in efficacy [31,197]. 

The composition of ASEDs may also be influenced by the patient’s underlying systemic or ocular disease. Serum from patients with autoimmune or systemic inflammatory disorders may differ from that of healthy individuals in the concentration of epitheliotropic and pro-inflammatory factors. These differences may be clinically relevant, especially in patients with systemic disease or poor response to ASED therapy [9,153].

Storage and handling are critical determinants of product stability and safety. The commonly used approach of storing the in-use vial at 4–8 °C and the remaining vials frozen at approximately −20 °C appears to preserve the measured concentrations of most growth factors. However, some neurotrophic peptides are less stable, which may be relevant for indications such as neurotrophic keratopathy. Microbial contamination remains a practical concern, particularly with repeated handling, although clinically significant infections are rarely reported. The use of single-day containers may reduce this risk and improve treatment safety [63,169,198].

The available evidence is limited by considerable methodological heterogeneity among studies, including differences in study design, preparation protocols, analytical methods, and reported parameters, which precluded quantitative synthesis. The review process was therefore based on a qualitative analysis of studies identified through the predefined database searches and the authors’ personal literature database. Limitations of the review process include the absence of a registered protocol and a formal risk-of-bias assessment, reflecting the broad descriptive scope and methodological heterogeneity of the included literature. Consequently, the findings should be interpreted as a descriptive synthesis of the available literature rather than as a comparative assessment of the quality or strength of evidence across individual studies, and they do not allow definitive conclusions regarding optimal preparation, storage, or safety protocols.

Overall, these findings emphasize that the biological efficacy of ASEDs depends not only on their composition but also on preparation, storage, and handling procedures. Future studies should use standardized and clearly reported protocols to generate more robust evidence on how individual preparation and storage parameters affect ASED composition, stability, and safety.

## 9. Conclusions

ASEDs represent a biologically active tear substitute containing a broad spectrum of epitheliotropic, neurotrophic, anti-inflammatory, antimicrobial, and other bioactive factors that may support ocular surface homeostasis and regeneration. Their biological composition resembles that of natural tears but may vary according to the patient’s underlying systemic and ocular conditions.

Despite substantial variability in preparation protocols, ASED production generally follows similar principles. However, differences in production, storage, and handling may affect the stability of individual bioactive components. In particular, some neurotrophic factors degrade more rapidly than other growth factors, which may be relevant for specific therapeutic indications.

Standardization of preparation, storage, and quality-control procedures may improve product consistency, safety, and reproducibility and facilitate comparison between clinical studies.

## Figures and Tables

**Table 1:** Comparison of human tear and serum composition in healthy controls and patients with DED: physicochemical parameters, proteins, and glycosaminoglycans.

Parameters	Healthy Control—Range of Means (Median *)		DED—Range of Means (Median *)	
	Tears	Serum	Tears	Serum
Osmolarity (mOsm/L)	298–302 [31,35,36,37,38,39,40]	296–300 [31,35]	296–334 [37,38,39,40]	
pH	7.4 [31,35]	7.2–7.5, 7.4 [31,35]		
Glucose (mg/mL)	0.026 [35]	0.6–1.2 [35]		
Total proteins (mg/mL)	**5.4–11.3** [35,41,42,43,44]	**68–82** [35,43]	6.4–6.9 [42,45]	
Albumin (mg/mL)	**0.05–1.3** [35,41,42,46,47]	**35–55** [35,48]	0.7–0.9 [42,45]	44 ± 2.9 [49]
Fibronectin (μg/mL)	**0.02–21** [31,41,50]	**140–205** [31,48]		300 ± 100 [49]
Hyaluronic acid (ng/mL)	115 (0–3020) [51]	15.5 (10–100) [52,53]		
Selenoprotein P (mg/mL)		4.5 *§ [54]		

**Table 2:** Comparison of human tear and serum composition in healthy controls and patients with DED: growth and neurotrophic factors.

Parameters	Healthy Control—Range of Means (Median *)		DED—Range of Means (Median *)	
	Tears	Serum	Tears	Serum
EGF (ng/mL)	0.09–5.1 [31,35,55,56,57,58,59]	0.2–0.8 [31,35,48,60,61,62,63]	0.06–2.3 [55,56,58]	0.19–0.26 [49,60]
TGF-β1 (ng/mL)	Not detected–**38.5** ** [35,64,65]	**6–140** [31,60,62,63,64,66,67,68,69,70]		37.7–74.7 [49,60,66]
TGF-β2 (ng/mL)	0.01–7.85 ** [35,65,71]	0.32–1.1 * [60,62,68]	0.014 [71]	0.55 ± 0.12 [60]
FGF (ng/mL)	0.01–0.02 [33,61]	0.0008 *–0.04 * [60,62,72]		0.007 [60]
PDGF-BB (ng/mL)	1.7 [64]	2.4 ± 0.6 [60]		3.3–65.5 [49,60]
HGF (ng/mL)	0.2–0.52 [73,74]	0.13–0.74 [60,72]		0.15 [60]
VEGF (ng/mL)	0.02–1.01 [44,72]	0.04–0.07 [60,62,69]		0.07–0.32 [49,60]
IGF-1 (ng/mL)	76 [75]	60–376 [14,60,63,75]		58.14 ± 16.76 [60]
NGF (pg/mL)	49 *–108 [14,75,76]	23.4 *–468.3 [14,60,67,75,77]		2.2–115 [49,60]
Substance P (pg/mL)	70 ± 25 [75]	157–170 [14,75]		
Neurotrophin 3 (pg/mL)	36 * [76]	110 ± 25.2 [77]		
Neurotrophin 4 (pg/mL)	23 * [76]	149 ± 48.7 [77]		
BDNF (ng/mL)	0.091 *, 0.113 [76,78]	10.15–26.4 [60,78]		8.81 [60]

**Table 3:** Comparison of human tear and serum composition in healthy controls and patients with DED: vitamins.

Parameters	Healthy Control—Range of Means (Median *)		DED—Range of Means (Median *)	
	Tears	Serum	Tears	Serum
A (Retinol) (ng/mL)	**0–16** [35,79]	**223–988** [35,48,61,79]		600 ± 200 [49]
B1 (Thiamine) (ng/mL)	2.9–10.6 [79]	2.1–8.5 [79]		
B2 (Riboflavin) (ng/mL)	4.1–30.1 [79]	6.0–23.7 [79]		
B3 (Nicotinamide) (ng/mL)	**68–928** [79]	**20–1588** [79]		
B5 (Pantothenic acid) (ng/mL)	81–241 [79]	24–162 [79]		
B9 (Folic acid) (ng/mL)	26–419 [79]	15–177 [79]		
C (Ascorbic acid) (µg/mL)	**39–231** [35,80,81]	**6.0–20** [35,82]		
D (total 25-hydroxyvitamin D) (ng/mL)	**3.2–45.8** (* 16.3) [83]	**3.3–27.5** (* 8.4) [83]	18.5 # [84]	12.8 # [84]
E (α-Tocopherol) (ng/mL)	**14–409** [79]	**1500–16,800** [61,79]		14,200 ± 3600 [49]

**Table 4:** Comparison of human tear and serum composition in healthy controls and patients with DED: antimicrobial agents and antioxidants.

Parameters	Healthy Control—Range of Means (Median *)		DED—Range of Means (Median *)	
	Tears	Serum	Tears	Serum
Immunoglobulin G (µg/mL)	**6.7–32** [35,43]	**8000–18,000** [35,43]		
IgA (µg/mL)	**186–410** [35,43,46]	**900–4500** [35,43,48,66]		2200–3400 * [49,66]
IgM (µg/mL)	**5.6** [43]	**370–2800** [35,43,66]		1550 * [66]
IgD (µg/mL)	Not detected [35,85]	**3–300** [35]		
IgE (µg/mL)	<0.0003–0.052 [86,87]	0.25–0.7 [35]		
Alpha-2 macroglobulin (µg/mL)	0.1 [88]	2.2 [88]		
Lysozyme (mg/mL)	**1.6–3.06** [35,42,46,47]	**0.001–0.015** [35]	**1.4–2.15** [42,45]	
Lactoferrin (mg/mL)	**1.51–2.73** [35,42,46,47,55]	30.7 ± 6.6 × 10^−5^ [89]	**0.13–1.64** [42,45,55]	
Lipocalin (mg/mL)	**1.23–2.95** [42,46,47]		**0.9–1.2** [42,45]	
Transferrin (mg/mL)	**0.9 ± 0.6** [42]	2.5 # [90]	**0.33 ± 0.3** [42]	
Tyrosine (µmol/L)	45–46 [35,91]	55–77 [35,92]		
Glutathione (µmol/L)	107 [35,91]	4–7 § [93]		
Superoxide dismutase activity (U/mL)	**442 ± 89** [94]	**89 ± 13** [94]		
Uric acid (µmol/L)	**119 ± 48** (cataract patients) [95]	**345 ±** 89 [95]		

Summary of the Table 1, Table 2, Table 3 and Table 4: Whenever possible, the selected values were obtained from studies directly comparing the concentrations of bioactive substances in tears and serum or between healthy and diseased individuals. Values are presented as means or ranges, unless otherwise specified (see footnotes); however, direct comparison of values derived from independent studies should be made with caution because study populations, sample collection methods, analytical methods, and reporting formats differed among studies. * Median, ** Concentrations TGF-β in acidified human tears (e.g., mature TGF), § Plasma value, # Read from the graph. Bold: statistically significant differences between tears and serum within the same study. BDNF: brain-derived neurotrophic factor; EGF: epidermal growth factor; FGF: fibroblast growth factor; HGF: hepatocyte growth factor; Ig: immunoglobulin; IGF-1: insulin-like growth factor I; NGF: nerve growth factor; PDGF-BB: platelet-derived growth factor-BB; TGF-β1: transforming growth factor-beta 1; TGF-β2: transforming growth factor-beta 2; VEGF: vascular endothelial growth factor.

**Table 5:** Stability of epitheliotropic and neurotrophic factors in ASEDs as a function of storage temperature and duration.

Study	AS (%)/ Solvent	Analytes	Storage (°C)	Storage (h, d, w, m)	Results	Method	Ref.
Tsubota 1999	100	EGF, TGF-β1, vitamin A	4	1 w, 1 m	Non-significant change	EGF: RIA; vitamin A: HPLC; TGF-β1: ELISA	[6]
			−20	1 and 3 m			
Tsubota 1999	20/PS	EGF, TGF-β1, vitamin A	4	1 w, 1 m	Stable	EGF: RIA; vitamin A: HPLC; TGF-β1: ELISA	[16]
			−20	1 and 3 m			
Leite 2006		Insulin and proteins	−20	1 m	Stable	RIA and biuret dye assay	[191]
Bradley 2009	20/BSS	TGF-β1, SubP, NGF, CGRP, IGF-1, EGF	4	3, 6, 12, 24 h	SubP significantly reduced after 24 h	ELISA	[169]
			25	1, 3, 6, 24 h	CGRP significantly reduced after 6 h and 24 h, SubP and NGF significantly reduced after 24 h		
			37	0.5, 1, 3, 9 h	Unstable after 3 h (SubP, NGF, CGRP), and 9 h (TGF-β1)		
			42	0.5, 1, 3, 9 h	Unstable after 3 h (TGF-β1, NGF, CGRP), and 9 h (SubP)		
			−15	1, 2, 3, 6 w	CGRP significantly reduced after 6 w		
Phasukkijwatana 2011	20/BSS	EGF, TGF-β1/2, fibronectin	4	1 w, 1 m	Stable	ELISA	[68]
			−20	1, 3, 6 m	Significant decrease in fibronectin (from 6 m); significant decrease in TGF-β1/2 (starting from 1 m)		
Fischer 2012	100/50/20/BSS	EGF, fibronectin, vitamins A and E, albumin, IgA	6	7 d	Stable	EGF, fibronectin: ELISA; vitamins: HPLC; albumin: photometry; IgA: turbidimetry	[48]
			−20	3, 6 m			
Lopez-Garcia 2014	50/PS/SH	EGF, TGF-β	4	1 m	Stable	ELISA	[123]
			−20	1, 2, 3 m			
Lopez-Garcia 2016	50/PS/SH	EGF, TGF-β1, PDGF-AB, albumin	4	1, 2, 3, 4 w	Stable	ELISA	[193]
			−20	1, 3, 6, 9 m			
Metheetrairut 2022	20/BSS	EGF, TGF-β1, fibronectin, PDGF-AB	4	1 w	Stable	ELISA	[105]
			−20	1, 3 m			
Jirsova 2022	100/20/PS	TGF-β1, EGF, IGF-1	4–8	24 h, 72 h, 7 and 8 d	TGF-β1 in diluted serum was stable.	ELISA	[63]
					TGF-β1 in concentrated serum decreased to 76% at 4–8 °C after 8 days; TGF-β1, EGF and IGF-1 were stable in 20% up to 1 week at 4–8 °C		
			−20	4 and 7 m	TGF-β1 and EGF in 20% serum were stable after 4 months and 7 months at −20, −80, and −156 °C; IGF-1 significantly decreased after 4 months		
			−80	4 and 7 m			
			−156	4 and 7 m			
Dormann 2024	100	TGF-β1, vitamin A, VEGF-A	−20	6 m	Stable	TGF-β1, VEGF-A: ELISA; vitamin A: HPLC;	[162]
Sharma 2025	50/SH, 50/CsA	EGF, TGF-β	4	2 w, 4 w	Stable up to 1 month	ELISA	[178]

h: hour; d: day; w: week; m: month; AS: autologous serum; BSS: balanced salt solution; CGRP: calcitonin gene-related peptide; CsA: cyclosporine 0.05%; EGF: epidermal growth factor; ELISA: enzyme-linked immunosorbent assay; HPLC: high-performance liquid chromatography; IGF-1: insulin-like growth factor-1; NGF: nerve growth factor; PS: physiological saline; RIA: radioimmunoassay; SH: sodium hyaluronate; SubP: substance P; TGF-β1: transforming growth factor β1; VEGF-A: vascular endothelial growth factor A.

## Data Availability

No new data were created or analyzed in this study. Data sharing is not applicable to this article.

## Supplementary Materials

The following supporting information can be downloaded at: https://www.mdpi.com/article/10.3390/bioengineering13101216/s1, Supplementary Table S1: History and Search Details, PubMed, Web of Science; Supplementary Table S2: List of studies included in the systematic review; Supplementary Table S3: Microbial contamination of ASED during preparation, storage, and use.

## Abbreviations

The following abbreviations are used in this manuscript:

ASEDs	Autologous serum eye drops
DED	Dry eye disease
EGF	Epidermal growth factor
HGF	Hepatocyte growth factor
IFN-γ	Interferon-γ
Ig	Immunoglobulin
IGF	Insulin-like growth factor
IL	Interleukin
MMP	Matrix metalloproteinases
NGF	Nerve growth factor
oGVHD	Ocular graft versus host disease
OSDI	Ocular surface disease index
PDGF	Platelet derived growth factor
PED	Persistent epithelial defects
SJS	Stevens-Johnson syndrome
TGF-β	Transforming growth factor beta
TNF-α	Tumor necrosis factor α
VEGF	Vascular endothelial growth factor
