# Supplementary material for: Autologous Serum Eye Drops: A Systematic Review, Part I—Characteristics, Preparation, Storage, and Stability
Source: Bioengineering (Basel). 2026 Oct 19;13(10):1216. doi: 10.3390/bioengineering13101216 (PMC13701193; doi:10.3390/bioengineering13101216)
Supplement: Supplementary file 1 [file bioengineering-13-01216-s001.zip › ASEDP1_Supplementary Table_S1.pdf]

History and Search Details, PubMed, Web of Science

#20			Search: **#4 OR #5 OR #6 OR #7 OR #8 OR #9 OR #10 OR #11 OR #12 OR #13 OR #14 OR #15 OR #16 OR #17 OR #18 OR #19** Sort by: **Most Recent**	276	06:39:30
#19			Search: **autologous serum[Title/Abstract] AND eye[Title/Abstract] AND ocular surface[Title/Abstract] AND humans[Filter]** Sort by: **Most Recent**	155	06:37:54
#18			Search: **autologous serum[Title/Abstract] AND eye[Title/Abstract] AND dry eye[Title/Abstract] AND humans[Filter]** Sort by: **Most Recent**	172	06:37:09
#17			Search: **autologous serum[Title/Abstract] AND eye[Title/Abstract] AND ("disease"[Title/Abstract] OR "disorder"[Title/Abstract]) AND humans[Filter]** Sort by: **Most Recent**	157	06:36:31
#16			Search: **autologous serum[Title/Abstract] AND eye[Title/Abstract] AND review[Title/Abstract] AND humans[Filter]** Sort by: **Most Recent**	64	06:36:07
#15			Search: **autologous serum[Title/Abstract] AND eye[Title/Abstract] AND ("effect"[Title/Abstract] OR "effects "[Title/Abstract]) AND humans[Filter]** Sort by: **Most Recent**	101	06:35:25
#14			Search: **autologous serum[Title/Abstract] AND eye[Title/Abstract] AND ("vitamin"[Title/Abstract] OR "cytokine"[Title/Abstract]) AND humans[Filter]** Sort by: **Most Recent**	20	06:34:49

#13			Search: **autologous serum[Title/Abstract] AND eye[Title/Abstract] AND ("growth factor"[Title/Abstract] OR "growth factors"[Title/Abstract]) AND humans[Filter]** Sort by: **Most Recent**	52	06:34:24
#12			Search: **autologous serum[Title/Abstract] AND eye[Title/Abstract] AND composition[Title/Abstract] AND humans[Filter]** Sort by: **Most Recent**	8	06:34:02
#11			Search: **autologous serum[Title/Abstract] AND eye[Title/Abstract] AND stability[Title/Abstract] AND humans[Filter]** Sort by: **Most Recent**	19	06:32:29
#10			Search: **autologous serum[Title/Abstract] AND eye[Title/Abstract] AND ("microbiological"[Title/Abstract] OR "contamination"[Title/Abstract]) AND humans[Filter]** Sort by: **Most Recent**	17	06:31:46
#9			Search: **autologous serum[Title/Abstract] AND eye[Title/Abstract] AND ("storage"[Title/Abstract] OR "preservation"[Title/Abstract]) AND humans[Filter]** Sort by: **Most Recent**	19	06:31:04
#8			Search: **autologous serum[Title/Abstract] AND eye[Title/Abstract] AND ("storage"[Title/Abstract] OR "freezing"[Title/Abstract]) AND humans[Filter]** Sort by: **Most Recent**	17	06:30:36
#7			Search: **autologous serum[Title/Abstract] AND eye[Title/Abstract] AND storage[Title/Abstract] AND humans[Filter]** Sort by: **Most Recent**	17	06:29:37
#6			Search: **autologous serum[Title/Abstract] AND eye[Title/Abstract] AND ("centrifugation"[Title/Abstract] OR "dilution"[Title/Abstract] OR "serum concentration"[Title/Abstract]) AND humans[Filter** Sort by: **Most Recent**	12	06:27:50
#5			Search: **autologous serum[Title/Abstract] AND eye[Title/Abstract] AND preparation[Title/Abstract] AND humans[Filter]** Sort by: **Most Recent**	25	06:26:45

#4			Search: **autologous serum[Title/Abstract] AND eye[Title/Abstract] AND history[Title/Abstract] AND humans[Filter]** Sort by: **Most Recent**	24	06:26:19
#3			Search: **autologous serum[Title/Abstract] AND eye[Title/Abstract] AND ("eye"[Title/Abstract] OR "cornea"[Title/Abstract] OR "conjunctiva"[Title/Abstract]) AND humans[Filter]** Sort by: **Most Recent**	305	06:23:14
#2			Search: **autologous serum[Title/Abstract] AND eye[Title/Abstract] AND humans[Filter]** Sort by: **Most Recent**	305	06:22:37
#1			Search: **autologous serum[Title/Abstract] AND eye[Title/Abstract]** Sort by: **Most Recent**		

Download
Delete

Search	Actions	Details	Query	Results	Time
#2			Search: **autologous serum[Title/Abstract] AND eye[Title/Abstract] AND (tear[Title/Abstract] OR tears[Title/Abstract] OR "tear film"[Title/Abstract] OR "tear composition"[Title/Abstract] OR "tear proteins"[Title/Abstract] OR cytokine[Title/Abstract] OR cytokines[Title/Abstract] OR "growth factor"[Title/Abstract] OR "growth factors"[Title/Abstract] OR vitamin[Title/Abstract] OR vitamins[Title/Abstract] OR antioxidant[Title/Abstract] OR antioxidants[Title/Abstract] OR antioxidative[Title/Abstract] OR antimicrobial[Title/Abstract] OR antimicrobials[Title/Abstract]) AND humans[Filter]** Sort by: **Computed Author**	193	07:10:29
#1			Search: **autologous serum[Title/Abstract] AND eye[Title/Abstract] AND (tear[Title/Abstract] OR tears[Title/Abstract] OR "tear film"[Title/Abstract] OR "tear composition"[Title/Abstract] OR "tear proteins"[Title/Abstract] OR cytokine[Title/Abstract] OR cytokines[Title/Abstract] OR "growth factor"[Title/Abstract] OR "growth factors"[Title/Abstract] OR vitamin[Title/Abstract] OR vitamins[Title/Abstract] OR antioxidant[Title/Abstract] OR antioxidants[Title/Abstract] OR antioxidative[Title/Abstract] OR antimicrobial[Title/Abstract] OR antimicrobials[Title/Abstract]) AND humans[Filter]** Sort by: **First Author**	193	06:52:36

8 September 2026

Web of science

Web of Science příkaz (Query Builder)	Number of results
(TS=("autologous serum") AND TS=(eye) AND TS=(history) AND TS=(human*)) OR (TS=("autologous serum") AND TS=(eye) AND TS=(preparation) AND TS=(human*)) OR (TS=("autologous serum") AND TS=(eye) AND TS=(centrifugation OR dilution OR "serum concentration") AND TS=(human*)) OR (TS=("autologous serum") AND TS=(eye) AND TS=(storage) AND TS=(human*)) OR (TS=("autologous serum") AND TS=(eye) AND TS=(storage OR freezing) AND TS=(human*)) OR (TS=("autologous serum") AND TS=(eye) AND TS=(storage OR preservation) AND TS=(human*)) OR (TS=("autologous serum") AND TS=(eye) AND TS=(microbiological OR contamination) AND TS=(human*)) OR (TS=("autologous serum") AND TS=(eye) AND TS=(stability) AND TS=(human*)) OR (TS=("autologous serum") AND TS=(eye) AND TS=(composition) AND TS=(human*)) OR (TS=("autologous serum") AND TS=(eye) AND TS=("growth factor" OR "growth factors") AND TS=(human*)) OR (TS=("autologous serum") AND TS=(eye) AND TS=(vitamin OR cytokine) AND TS=(human*)) OR (TS=("autologous serum") AND TS=(eye) AND TS=(effect OR effects) AND TS=(human*)) OR (TS=("autologous serum") AND TS=(eye) AND TS=(review) AND TS=(human*)) OR (TS=("autologous serum") AND TS=(eye) AND TS=(disease OR disorder) AND TS=(human*)) OR (TS=("autologous serum") AND TS=(eye) AND TS=("dry eye") AND TS=(human*)) OR (TS=("autologous serum") AND TS=(eye) AND TS=("ocular surface") AND TS=(human*))	131
TS=("autologous serum") AND TS=(eye) AND TS=("ocular surface") AND TS=(human*)	82
TS=("autologous serum") AND TS=(eye) AND TS=("dry eye") AND TS=(human*)	76

TS=("autologous serum") AND TS=(eye) AND TS=(disease OR disorder) AND TS=(human*)	86
TS=("autologous serum") AND TS=(eye) AND TS=(review) AND TS=(human*)	29
TS=("autologous serum") AND TS=(eye) AND TS=(effect OR effects) AND TS=(human*)	59
TS=("autologous serum") AND TS=(eye) AND TS=(vitamin OR cytokine) AND TS=(human*)	20
TS=("autologous serum") AND TS=(eye) AND TS=("growth factor" OR "growth factors") AND TS=(human*)	81
TS=("autologous serum") AND TS=(eye) AND TS=(composition) AND TS=(human*)	6
TS=("autologous serum") AND TS=(eye) AND TS=(stability) AND TS=(human*)	12
TS=("autologous serum") AND TS=(eye) AND TS=(microbiological OR contamination) AND TS=(human*)	6
TS=("autologous serum") AND TS=(eye) AND TS=(storage OR preservation) AND TS=(human*)	12
TS=("autologous serum") AND TS=(eye) AND TS=(storage OR freezing) AND TS=(human*)	15
TS=("autologous serum") AND TS=(eye) AND TS=(storage) AND TS=(human*)	11
TS=("autologous serum") AND TS=(eye) AND TS=(centrifugation OR dilution OR "serum concentration") AND TS=(human*)	7

TS=("autologous serum") AND TS=(eye) AND TS=(preparation) AND TS=(human*)	15
TS=("autologous serum") AND TS=(eye) AND TS=(history) AND TS=(human*)	5
TS=("autologous serum") AND TS=(eye) AND (TS=(eye) OR TS=(cornea) OR TS=(conjunctiva)) AND TS=(human*)	132
TS=("autologous serum") AND TS=(eye) AND TS=(human*)	132
TS=("autologous serum") AND TS=(eye)	828
TS=("autologous serum") AND TS=(eye OR ocular) AND TS=(human*)	155 – for PRISMA identification

11September 2026
