# Supplementary material for: Autologous Serum Eye Drops: A Systematic Review, Part I—Characteristics, Preparation, Storage, and Stability
Source: Bioengineering (Basel). 2026 Oct 19;13(10):1216. doi: 10.3390/bioengineering13101216 (PMC13701193; doi:10.3390/bioengineering13101216)
Supplement: Supplementary file 1 [file bioengineering-13-01216-s001.zip › ASEDP1_Supplementary_Table_S3_v01.pdf]

ASEDP1_Supplementary_Table_S3 Microbial contamination of ASED during preparation, storage, and use.

Study	Setting / N	ASED / container	Storage / use	Microbiological testing (media; incubation)	Positive results, n/N (%)
**CLINICAL USE — in-use contamination**					
Lagnado_2004	Inpatient; 14P; 67 bottle-days	20%; 2 ml dropper	4°C; 24 h/bottle; pre/post use	Blood agar, broth; 37°C/48 h	Pre 3/67 (4.5%); post 10/67 (14.9%); total 13/134 (9.7%)
Leite_2006	Patient use; 12P; 11 samples	NR; dropper	Reserve frozen; in-use refrigerated; sampled d30 after preparation	B/F cultures; conditions NR	6/11 (54.5%)
López-García_2012	Home: 48 filtered, 128 conventional; inpatient: 14	20%; filter-equipped dropper (0.22 μm, SH) / standard dropper (saline)	4°C; home: 1–4 w; inpatient: to 4 w	Blood, chocolate, Sabouraud agar; B 37°C/48 h; F 5 d	Filtered 1/48 (2.1%), 4 w; standard 37/128 (28.9%), 1 w; inpatient 7/14 (50%), 4 w
Thanathanee_2013	Inpatient; 21P; 147 bottles	100%; 2 ml dropper	4°C; 24 h/bottle; pre/post use	Blood, chocolate, MacConkey, Sabouraud; ≥6 w	Pre 11/147 (7.5%); post 7/147 (4.8%); total 18/294 (6.1%)
Sanak_2026	Home; 17P; 229 glass/104 valve	Glass / one-way valve dropper	Mean use: glass 3.8 d; valve 4.1 d	Tip: chocolate agar; contents: thioglycollate; incubation NR	Glass contents: 50% at 2.5 d*; valve: 1/104 (1.0%)
**CONTROLLED TESTING — manufacture, storage, simulated use**					
Prabhasawat_2005	Storage; 5 samples	20%; bottle	4°C; to d84; unused	Tryptic soy, thioglycollate broths; NR	No confirmed growth
Reed-Kane_2009	Storage; 10 syringes	20%; sealed syringes	2–8°C to 30 d; −10°C to 180 d	USP sterility test; details NR	No growth
Blasetti_2015	Laboratory; 100/300 cultures	100%; 0.5 ml syringes	4°C to d4; q2h simulation	Chocolate, Sabouraud; 37°C/96 h	Storage 0/100; simulated use 0/300 cultures
Yang_2020	Lab; 100P; 800 cultures/arm	20% alone / +Dex / +Lev; 5 ml bottles	4°C/28 d; opened 3–4×/d	Blood, chocolate, Sabouraud; 37°C/≥4 w; closed culture d28	Open B: 38, 27, 7/800; F: 2, 2, 3/800; closed B: 2, 1, 0/100
Dormann_2024	GMP; 9P/11 donations; n=8 at 6 m	100%; 1.5 ml single-use	−20±2°C; 6 m	BACTEC aerobic/anaerobic; 30–32°C/7 d	No growth; 6-m subset n=8
Blaser_2026	Microbial challenge; 11 strains	50%/100%; 2.5 ml ophtioles	2–8°C/20–25°C; up to 48 h	SA/COS/SGA; microbial enumeration 0–48 h	No increase: 11/11 strains at 2–8°C; 7/11 at RT

Notes: ASED, autologous serum eye drops; P, patients; B/F, bacterial/fungal; Dex, dexamethasone; Lev, levofloxacin; NR, not reported; d, days; w, weeks; m, months; RT, room temperature. Denominators represent bottles, cultures or batches; rates are not directly comparable. Pre/post: before/after 24 h of use.
Sanak_2026: 50% is time-specific, not an overall bottle rate. López-García_2012: different diluents and use periods. Yang_2020: results in no-additive/Dex/Lev order; repeated cultures; closed test at d28. Blaser_2026: microbial challenge, not contamination incidence.

1. Lagnado R, King AJ, Donald F, Dua HS. A protocol for low contamination risk of autologous serum drops in the management of ocular surface disorders. Br J Ophthalmol. 2004;88(4):464-465. doi:10.1136/bjo.2003.025528. PMID: 15031155.
2. Leite SC, de Castro RS, Alves M, Cunha DA, Correa ME, da Silveira LA, et al. Risk factors and characteristics of ocular complications, and efficacy of autologous serum tears after haematopoietic progenitor cell transplantation. Bone Marrow Transplant. 2006;38(3):223-227. doi:10.1038/sj.bmt.1705426. PMID: 16785864.
3. López-García JS, García-Lozano I. Use of containers with sterilizing filter in autologous serum eyedrops. Ophthalmology. 2012;119(11):2225-2230. doi:10.1016/j.ophtha.2012.06.028. PMID: 22867978.

4. Thanathanee O, Phanphruk W, Anutarapongpan O, Romphruk A, Suwan-Apichon O. Contamination risk of 100% autologous serum eye drops in management of ocular surface diseases. Cornea. 2013;32(8):1116-1119. doi:10.1097/ICO.0b013e3182910036. PMID: 23665646.
5. Sanak F, Iselin K, Kaufmann C, Tanadini M, Arnold M, Barbanti L, et al. Optimized autologous serum eye drops: microbial contamination after extended length of use of two different types of eye dropper bottles. Cornea. 2026;45(10):1240-1245. doi:10.1097/ICO.0000000000004023. PMID: 41233984.
6. Prabhasawat P, Chotikavanich S, Leelaporn A. Sterility of non-preservative eye drops. J Med Assoc Thai. 2005;88 Suppl 9:S6-S10. PMID: 16681044.
7. Reed-Kane D, Carlson RA, Kupiec TC, Vu N. Applications and sterility of autologous serum eye drops. Int J Pharm Compd. 2009;13(6):540-543. PMID: 23965328.
8. Blasetti F, Usai D, Sotgia S, Carru C, Zanetti S, Pinna A. A protocol for microbiologically safe preparation, storage, and use of autologous serum eye-drops in low-income countries. J Infect Dev Ctries. 2015;9(1):55-59. doi:10.3855/jidc.4733. PMID: 25596572.
9. Yang Y, Zhu X, Yang J, Shi A, Jiang M, Peng Y, et al. An environmental control experiment for contamination of the production and storage of 20% autologous serum eye drops. Curr Eye Res. 2020;45(11):1364-1368. doi:10.1080/02713683.2020.1741008. PMID: 32150475.
10. Dormann F, Hähnel V, Müller V, Burkhardt R, Offner R. Allogenic and autologous nondiluted serum eye drops-validation strategy compliant with good manufacturing practice. Transfus Med. 2024;34(4):319-325. doi:10.1111/tme.13068. PMID: 39037018.
11. Blaser F, Barthelmes D, Korner G, Bugajska J, Nolte O, Zweifel SA, et al. Microbial assessment of reclosable single-day use ophtioles containing autologous serum eye drops. Bioengineering (Basel). 2026;13(4):417. doi:10.3390/bioengineering13040417. PMID: 42072211.
